# Supplementary material for: Boiler: lossy compression of RNA-seq alignments using coverage vectors
Source: Nucleic Acids Res. 2016 Jun 13;44(16):e133. doi: 10.1093/nar/gkw540 (PMC5027496; doi:10.1093/nar/gkw540)
Supplement: SUPPLEMENTARY DATA [file supp_44_16_e133__index.html]

Boiler: lossy compression of RNA-seq alignments using coverage vectors — SUPPLEMENTARY DATA 

# Boiler: lossy compression of RNA-seq alignments using coverage vectors

## SUPPLEMENTARY DATA

- SUPPLEMENTARY DATA
