# Supplementary material for: Boiler: lossy compression of RNA-seq alignments using coverage vectors
Source: Nucleic Acids Res. 2016 Jun 13;44(16):e133. doi: 10.1093/nar/gkw540 (PMC5027496; doi:10.1093/nar/gkw540)
Supplement: SUPPLEMENTARY DATA [file supp_gkw540_nar-00651-met-n-2016-File009.pdf]

Supplementary Data for Boiler: Lossy compression of RNA-seq alignments using coverage vectors

Jacob Pritt [1,2*], Ben Langmead [1,2*]

[1]Department of Computer Science, Johns Hopkins University, Baltimore, MD [2]Center for Computational Biology, Johns Hopkins University, Baltimore, MD
* Correspondence to: Jacob Pritt, jacobpritt@gmail.com and Ben Langmead, langmea@cs.jhu.edu

SUPPLEMENTARY NOTE 1

LEMMA 0.1. *The read decompression problem is strongly NP-hard.*

Proof Consider the Multiple Subset Sum Problem (MSSP), defined as follows. Given n items with weights $w_1,w_2,...,w_n$ and m knapsacks with capacities $c_1,c_2,...,c_m$, assign items such that:

1. each item is assigned to up to 1 knapsack
2. the capacity of each knapsack is not exceeded by the combined weights of the items assigned to it
3. the total weight of the items in all the knapsacks is maximized.

MSSP is known to be strongly NP-hard (1).

We reduce MSSP to a special case of the read decompression problem where the coverage vector never exceeds 1. We first construct a vector C encoding knapsack capacities in unary. We start with empty C then, for each i, append c_i 1s followed by a single 0. Because the length of C depends on the numeric knapsack weights, this is a pseudo-polynomial time reduction. Next, we let the read length tally equal the item weight tally. Finally, we run our decompression algorithm on the coverage vector C and read length tally. The algorithm packs reads into the nonzero stretches of C. This solution is converted to an MSSP solution by converting reads to the corresponding items and stretches of the coverage vector to the corresponding knapsacks.

The reduction satisfies the requirements of a pseudo-polynomial transformation (2). Hence, the read decompression problem for unpaired reads is strongly NP-hard. □

SUPPLEMENTARY NOTE 2

Greedy algorithm for obtaining reads from coverage vector.

The algorithm works from one end of the coverage vector to the other, removing reads that remain consistent with the coverage vector. We take advantage of the homogeneous read length distribution produced by sequencing experiments by preferentially removing reads of the most common length. When necessary, we adjust the lengths of previously found reads by a few bases to match the coverage vector as closely as possible.

Initially, we extract reads in end-to-end sets of the form (a,b,n) where a and b are the starting and ending indices in the coverage vector and n is the number of end-to-end reads. Each read set must satisfy

$$n \cdot l_{min} \leq (b-a) \leq n \cdot l_{max}$$

where l_{min} and l_{max} are the minimum and maximum lengths in the read distribution, respectively. Each time we find a new read (b,c), we search for an existing read set matching (a,b,n) and update it to $(a,c,n+1)$. If no such read exists, we add a new read set $(b,c,1)$.

We define two helper functions $extend(x_0,x_1)$ and $shorten(x_0,x_1)$.

$extend(x_0,x_1)$ searches for a read set of the form (a,x_0,n) satisfying

$$n \cdot l_{min} \leq (x_1-a) \leq n \cdot l_{max}$$

and updates it to (a,x_1,n) and decrements the coverage vector in the range $[x_0,x_1)$ by 1.

$shorten(x_0,x_1)$ searches for a read set of the form (a,x_1,n) satisfying

$$n \cdot l_{min} \leq (x_0-a) \leq n \cdot l_{max}$$

and updates it to (a,x_0,n) and increments the coverage vector in the range $[x_0,x_1)$ by 1.

These functions allow us to adjust previous reads by small amounts to fit in later reads. The read extraction algorithm works as follows:

Last $start$ and end be the indices of the first and last nonzero elements in the coverage vector, respectively. We find a and b such that $cov[i] > 0 \forall i \in [start,a)$, $cov[a] = 0$ and $cov[i] = 0 \forall i \in [a,b)$, $cov[b] > 0$.

Special end case: if $a = end < start + l_{min}$, we first attempt to run $extend(start,a)$. If unsuccessful, we decrement the bases in the coverage vector in the range $[start,a)$ but do not add a new read.

If $a \geq l_{mode}$, we add a new read $(start, start + l_{mode})$ and update the coverage vector.

© 2016 The Author(s)

Otherwise, we attempt to run $extend(start,a)$. If unsuccessful, we attempt to run $shorten(a,b)$. If this is also unsuccessful, we do one of the following:

1. If $(a-start)\geq l_{min}$, we add a new read $(start,a)$ and update the coverage vector.

2. If $\frac{l_{min}}{2}\leq(a-start)<l_{min}$, we add a new read $(start,start+l_{mode})$ and update the coverage vector.

3. If $(a-start)<\frac{l_{min}}{2}$, we decrement the bases in the coverage vector in the range $[start,a)$ but do not add a new read.

We then update $start$ and end and repeat until the coverage vector is empty.

SUPPLEMENTARY NOTE 3

Tool versions and parameter settings.

- TopHat 2 v2.1.0
- HISAT v0.1.6
- Boiler v1.0.1 (on PyPy 2.4)
- CRAMTools v3.0 (on Java v1.7)
- Goby v2.3.5 (on Java v1.7)
- Cufflinks v2.2.1
- StringTie v1.2.2
- SAMtools v0.1.19
- BEDtools v2.25.0

Boiler and CRAMTools were run with default options. Goby was run with in full "ACT H+T+D" mode as described in the "Goby parameter settings" section of the Goby study (**?**).

Boiler and Goby remove read names by default, but CRAM does not. CRAMtools has an option `--preserve-read-names`, but we cannot find a working mechanism in version 3 to remove read names. Thus, for a fairer comparison, we stripped the read names before compressing.

When running Cufflinks, we used the `--no-effective-length-correction` option to avoid variability due to an issue (recently resolved) in how Cufflinks performs effective transcript length correction. StringTie was run with default parameters.

A full listing of the parameter settings used for the evaluations is at the following URL:
`http://bit.ly/boiler-201605-expts`

SUPPLEMENTARY NOTE 4

Boiler Compression Ratio for HISAT Output

Table 2 compares the compression ratio and compressed size for alignment files generated by TopHat 2 and HISAT. The initial file size is the size of the BAM with read names removed. For most paired-end datasets the HISAT alignment BAM was larger than the TopHat 2 BAM, but the compressed files generated by Boiler were roughly the same size. This leads to a better compression ratio for HISAT alignments.

Goby Compression Ratio

In Table 4 and Figure 3 of our main paper, we compare Boiler's compression rate to CRAM and Goby. As summarized in Table 1 of the main paper, CRAM does not preserve quality scores and tags by default, and we further remove read names before compressing for a fair comparison. Goby discards read names and most tags, but preserves quality score information and 'MD' tag for mismatches. Goby also preserves the identity of orphaned reads, which Boiler converts to unpaired reads. Table 2 compares the effect on Goby's compression rate of (1) converting orphaned reads to unpaired reads by modifying the SAM flags field, and (2) replacing all quality scores with '#' for efficient compression. For all datasets, orphaned reads represent only a small fraction of Goby storage, and removing quality scores reduces compressed size by less than 5% in unpaired datasets and less than 3% in all paired-end datasets.

SUPPLEMENTARY NOTE 5

Though Boiler allows the user to pose targeted queries without decompressing the entire file, we also evaluate how long Boiler takes to decompress an entire file relative to other tools. These results are presented in Table 3. Overall, Boiler takes roughly 2 – 4 times longer than CRAMTools and about 1 – 2 times longer than Goby to decompress entire files.

Supplementary Table 1. Compression ratio and compressed size of Boiler for alignments generated by TopHat and HISAT.

Dataset	TopHat		HISAT	
	Ratio	Size (MB)	Ratio	Size
Drosophila, Simulated Unpaired				
0.5M	9.6	2.7	9.7	2.7
1M	13.6	3.6	14.0	3.6
2.5M	21.3	5.6	21.3	5.5
5M	29.1	7.6	29.8	7.5
10M	39.7	11.0	40.1	10.8
20M	55.7	15.0	56.7	14.8
Drosophila, Simulated Paired				
0.5M	13.1	4.3	14.8	4.3
1M	15.5	6.7	17.7	6.8
2.5M	19.8	12.9	22.4	12.9
5M	24.3	20.0	27.8	20.3
10M	30.2	31.6	34.8	31.6
20M	38.8	49.0	43.4	49.8
Human				
SRP025982 (11M)	21.3	38.0	21.9	65.9
Simulated 20M	29.6	71.6	37.0	61.9
Simulated 40M	32.7	118.0	44.0	99.7

SUPPLEMENTARY NOTE 6

We investigated the effect of varying the threshold k in the exon scoring equation (1) in the main paper. As k increases, the scoring function becomes more relaxed, allowing exons with more divergent boundaries to contribute to the score. If $k=0$, then two exons receive a score of 1 only if they are identical, 0 otherwise. On the other extreme, as $k\rightarrow\infty$ the score approaches 1 for all pairs of exons. Figure 1 shows the precision and recall at varying k thresholds for the simulated *D. melanogaster* dataset containing 1 million paired-end reads. We observe that, across various k cutoffs, accuracy decreases slightly after Boiler compression. Both plots show an inflection point around $k=5$ to 10, after which the accuracy measure becomes more stable. Based on these results, we used a threshold of $k=10$ in all experiments.

SUPPLEMENTARY NOTE 7

Table 4 shows the read-level precision and recall for the HISAT-generated alignments, before and after compression with Boiler.

SUPPLEMENTARY NOTE 8

Tables 5 and 6 show the transcript-level precision and recall not weighted by coverage, compared to the reference transcriptome. Tables 7 and 8 show the precision and recall not weighted by coverage for the direct comparison of transcriptomes before and after compression.

SUPPLEMENTARY NOTE 9

Weighted k-mer recall.

We assess fidelity by measuring weighted k-mer recall (WKR), a component of the KC score developed by Li et al. (3) to assess transcriptome assemblies. WKR measures the degree to which an assembly recovers k-mers from the true simulated transcriptome, weighted by abundances of simulated transcripts containing the k-mer. For a k-mer r, its frequency profile $p(r)$ is defined as:

$$p(r)=\frac{\sum_{t\in T}n(r,t)c(t)}{\sum_{t\in T}n(t)c(t)}$$

where T is the simulated transcriptome and for each transcript $t\in T$:

Supplementary Table 2. Size of Goby compressed files for the BAM file with orphaned read information removed, and for all quality scores replaced by constant values. All files were compressed with the H+D+T codec.

Dataset	Original BAM	No Orphans	No Quality Scores
Drosophila, Simulated Unpaired			
0.5M	1.2	–	1.2
1M	2.4	–	2.3
2.5M	5.6	–	5.4
5M	10.1	–	9.8
10M	19.0	–	18.2
20M	35.7	–	34.2
Drosophila, Simulated Paired			
0.5M	4.9	4.9	4.8
1M	9.4	9.3	9.3
2.5M	23.8	23.6	23.4
5M	45.9	45.6	45.1
10M	95.8	95.3	94.3
20M	193.3	192.3	190.2
Human			
SRP025982 (11M)	159.8	159.8	154.9
HG00100 (20M)	352.2	352.2	347.4
Simulated 20M	307.8	307.0	304.2
Simulated 40M	587.6	585.5	580.7

Supplementary Table 3. Decompression times in seconds.

Dataset	Boiler	CRAM	Goby
Drosophila, Simulated Unpaired			
0.5M	20.3	9.4	14.3
1M	23.3	14.3	20.5
2.5M	40.7	29.5	36.8
5M	49.8	50.0	64.1
10M	82.9	87.8	110.9
20M	186.7	168.2	240.3
Drosophila, Simulated Paired			
0.5M	31.7	16.1	23.0
1M	44.5	25.7	35.8
2.5M	97.5	55.4	70.8
5M	161.0	99.0	121.6
10M	344.5	192.6	239.2
20M	1089.4	378.4	519.8
Human			
SRP025982 (11M)	828.2	315.1	654.0
HG00100 (20M)	1203.5	462.0	–*
Simulated 20M	1008.8	464.0	986.4
Simulated 40M	3179.7	835.3	1552.0

* Received an error when decompressing.

Supplementary Table 4. HISAT precision and recall of SAM reads.

Dataset	Ignoring Pairings		Including Pairings	
	Precision	Recall	Precision	Recall
Drosophila, Simulated Unpaired				
0.5M	0.992	0.993	–	–
1M	0.988	0.990	–	–
2.5M	0.980	0.984	–	–
5M	0.973	0.978	–	–
10M	0.967	0.973	–	–
20M	0.969	0.974	–	–
Drosophila, Simulated Paired				
0.5M	0.990	0.992	0.504	0.505
1M	0.984	0.988	0.404	0.406
2.5M	0.974	0.979	0.298	0.300
5M	0.969	0.974	0.221	0.222
10M	0.967	0.972	0.173	0.174
20M	0.968	0.972	0.140	0.141
Human				
SRP025982 (11M)	0.991	0.991	0.321	0.321
Simulated 20M	0.975	0.979	0.250	0.251
Simulated 40M	0.976	0.980	0.235	0.236

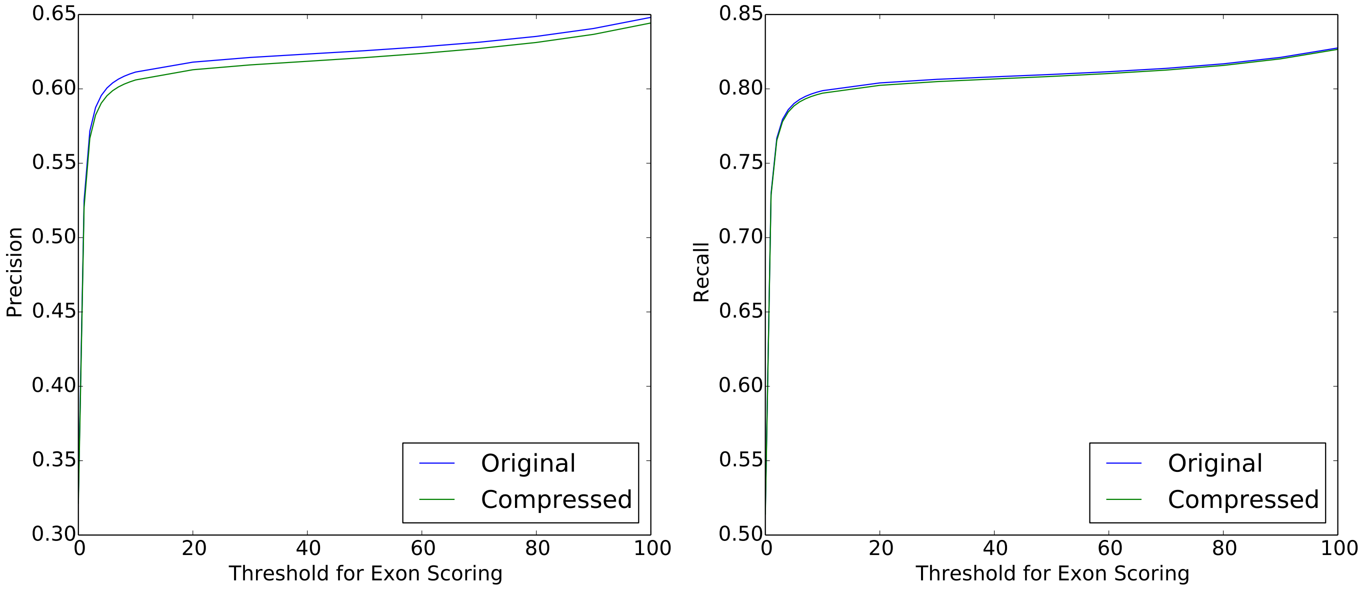

Supplementary Figure 1. Precision (left) and recall (right) of transcripts compared to the reference transcriptome before and after compression with Boiler, as a function of the threshold used in the exon scoring function.

- $n(r,t)$ is the number of times r occurs in t,
- $n(t)$ is the total number of k-mers in t, and
- $c(t)$ is the coverage of t.

Letting $R(T)$ be the set of all k-mers in transcriptome T:

$$WKR = \sum_{r \in R(T)} p(r)$$

WKR is defined with respect to the true transcriptome T, which we obtain from Flux Simulator's output. The GEUVADIS sample is not considered here, since it is not simulated. Figure 2 shows that WKR is largely unchanged after Boiler compression for various k-mer length settings.

It also shows that the difference in WKR is more pronounced for Cufflinks than for StringTie. Table 9 shows the WKR for $k=15$ for all datasets. Overall, the differences are slight, with the biggest difference at $k=15$ being an increase of 0.4% for the paired-end 2.5M-read *D. melanogaster* sample.

Supplementary Table 5. Reference-based Unweighted Precision.

Dataset	Cufflinks		StringTie	
	Original	Compressed	Original	Compressed
Drosophila, Simulated Unpaired				
0.5M	0.245	0.245 (+0.2%)	0.334	0.334 (+0.1%)
1M	0.278	0.278 (-0.1%)	0.392	0.392 (+0.0%)
2.5M	0.289	0.289 (-0.0%)	0.415	0.415 (+0.0%)
5M	0.274	0.274 (-0.0%)	0.379	0.379 (+0.0%)
10M	0.255	0.255 (+0.1%)	0.347	0.347 (+0.0%)
20M	0.246	0.247 (+0.2%)	0.320	0.320 (-0.1%)
Drosophila, Simulated Paired				
0.5M	0.402	0.400 (-0.4%)	0.389	0.388 (-0.1%)
1M	0.388	0.388 (-0.2%)	0.404	0.403 (-0.2%)
2.5M	0.361	0.361 (-0.0%)	0.384	0.383 (-0.1%)
5M	0.327	0.326 (-0.4%)	0.352	0.352 (-0.0%)
10M	0.299	0.300 (+0.1%)	0.321	0.321 (-0.0%)
20M	0.285	0.286 (+0.1%)	0.302	0.300 (-0.5%)
Human, Simulated Paired				
20M	0.204	0.203 (-0.4%)	0.214	0.213 (-0.3%)
40M	0.190	0.190 (-0.1%)	0.197	0.196 (-0.7%)

Supplementary Table 6. Reference-based Unweighted Recall

Dataset	Cufflinks		StringTie	
	Original	Compressed	Original	Compressed
Drosophila, Simulated Unpaired				
0.5M	0.367	0.368 (+0.0%)	0.266	0.266 (+0.1%)
1M	0.566	0.565 (-0.2%)	0.496	0.496 (+0.0%)
2.5M	0.717	0.716 (-0.1%)	0.708	0.708 (+0.0%)
5M	0.768	0.768 (+0.0%)	0.778	0.778 (-0.0%)
10M	0.784	0.784 (+0.0%)	0.806	0.806 (-0.0%)
20M	0.783	0.783 (-0.1%)	0.811	0.811 (-0.0)
Drosophila, Simulated Paired				
0.5M	0.573	0.571 (-0.3%)	0.480	0.480 (-0.1%)
1M	0.698	0.697 (-0.2%)	0.666	0.666 (-0.1%)
2.5M	0.772	0.774 (+0.2%)	0.785	0.784 (-0.0%)
5M	0.796	0.794 (-0.2%)	0.816	0.815 (-0.1%)
10M	0.783	0.778 (-0.6%)	0.806	0.805 (-0.0%)
20M	0.793	0.793 (+0.0%)	0.812	0.812 (-0.0%)
Human, Simulated Paired				
20M	0.748	0.747 (-0.2%)	0.777	0.775 (-0.3%)
40M	0.747	0.744 (-0.3%)	0.779	0.775 (-0.5%)

SUPPLEMENTARY NOTE 10

Tripartite Score

We developed a different scoring method to compare the accuracy of alignments before and after compression, called

Supplementary Table 7. Non-reference-based unweighted precision. Columns labeled Boiler compare precision before and after Boiler compression. Columns labeled Tech Reps compare pairs of technical replicates.

Dataset	Cufflinks		Stringtie	
	Boiler	Tech Reps (min–max)	Boiler	Tech Reps (min–max)
Drosophila, Simulated Unpaired				
0.5M	0.999	0.246–0.255	1.000	0.295–0.307
1M	0.998	0.322–0.333	1.000	0.385–0.398
2.5M	0.996	0.430–0.437	0.999	0.521–0.534
5M	0.996	0.504–0.512	0.999	0.607–0.613
10M	0.994	0.570–0.577	0.997	0.676–0.686
20M	0.990	0.629–0.634	0.994	0.725–0.733
Drosophila, Simulated Paired				
0.5M	0.981	0.425–0.440	1.000	0.389–0.405
1M	0.980	0.522–0.529	0.999	0.490–0.499
2.5M	0.969	0.624–0.632	0.996	0.613–0.619
5M	0.967	0.674–0.684	0.995	0.680–0.690
10M	0.960	0.713–0.722	0.992	0.727–0.733
20M	0.953	0.746–0.754	0.989	0.763–0.773
Human				
SRP025982 (11M)	0.971	0.384–0.389	0.999	0.314–0.318
HG00100 (20M)	0.921	(no replicates)	0.993	(no replicates)
Simulated 20M	0.969	0.761–0.786	0.991	0.756–0.787
Simulated 40M	0.964	0.780–0.804	0.986	0.777–0.809

Supplementary Table 8. Non-reference-based unweighted recall. Columns labeled Boiler compare recall before and after Boiler compression. Columns labeled Tech Reps compare pairs of technical replicates.

Dataset	Cufflinks		Stringtie	
	Boiler	Tech Reps (min–max)	Boiler	Tech Reps (min–max)
Drosophila, Simulated Unpaired				
0.5M	0.997	0.246–0.255	1.000	0.295–0.307
1M	0.995	0.322–0.333	1.000	0.385–0.398
2.5M	0.996	0.430–0.437	0.999	0.521–0.534
5M	0.995	0.504–0.512	0.999	0.607–0.613
10M	0.993	0.570–0.577	0.997	0.676–0.686
20M	0.988	0.629–0.634	0.995	0.725–0.733
Drosophila, Simulated Paired				
0.5M	0.981	0.425–0.440	0.999	0.389–0.405
1M	0.981	0.522–0.529	0.999	0.490–0.499
2.5M	0.972	0.624–0.632	0.996	0.613–0.619
5M	0.970	0.674–0.684	0.995	0.680–0.690
10M	0.959	0.713–0.722	0.992	0.727–0.733
20M	0.953	0.746–0.754	0.989	0.763–0.773
Human				
SRP025982 (11M)	0.964	0.384–0.389	0.999	0.314–0.318
HG00100 (20M)	0.922	(no replicates)	0.993	(no replicates)
Simulated 20M	0.968	0.761–0.786	0.992	0.756–0.787
Simulated 40M	0.961	0.780–0.804	0.987	0.777–0.809

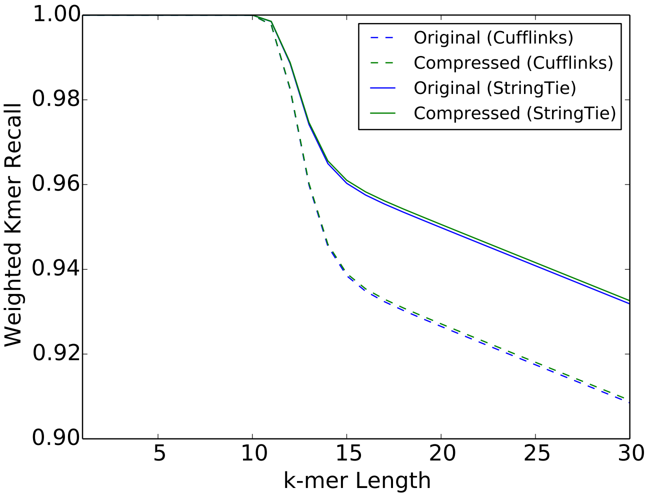

Supplementary Figure 2. WKR with varying k-mer length for simulated Drosophila 10M paired-end reads, assembled with Cufflinks and StringTie.

the tripartite score. There are two versions of this score, strict and loose.

We first construct a tripartite graph containing a node for each transcript in the cufflinks output for the alignments both before and after compression, as well as for each transcript in the reference transcriptome. We add a connecting edge from each transcript from the original set to the best-matching transcript from the reference set, determined using the transcript scoring method described previously. Similarly, we add an edge from each transcript in the compressed set to the best match from the reference set of transcripts.

For the strict tripartite score, we take all the nodes from the set of reference transcripts that are connected to a single node A_i from the set of original transcripts and a single node B_i

Supplementary Table 9. WKR.

Dataset	Cufflinks		StringTie	
	Original	Compressed	Original	Compressed
Drosophila, Simulated Unpaired				
0.5M	0.738	0.737 (-0.1%)	0.621	0.621 (+0.0%)
1M	0.857	0.856 (-0.0%)	0.776	0.776 (+0.0%)
2.5M	0.924	0.922 (-0.2%)	0.897	0.897 (+0.0%)
5M	0.949	0.949 (-0.0%)	0.935	0.935 (+0.0%)
10M	0.957	0.958 (+0.1%)	0.954	0.955 (+0.0%)
20M	0.962	0.961 (-0.0%)	0.960	0.960 (+0.0%)
Drosophila, Simulated Paired				
0.5M	0.848	0.848 (+0.0%)	0.779	0.779 (-0.0%)
1M	0.909	0.908 (-0.1%)	0.881	0.881 (-0.0%)
2.5M	0.929	0.932 (+0.4%)	0.940	0.941 (+0.0%)
5M	0.948	0.945 (-0.3%)	0.956	0.956 (+0.0%)
10M	0.938	0.933 (-0.6%)	0.960	0.961 (+0.1%)
20M	0.936	0.936 (-0.0%)	0.964	0.965 (+0.1%)
Human, Simulated				
20M	0.881	0.883 (+0.1%)	0.933	0.933 (+0.0%)
40M	0.900	0.908 (+0.8%)	0.934	0.934 (+0.1%)

from the set of compressed transcripts. The final score is the average of the transcript scores for every pair A_i, B_i.

For the loose tripartite score, we take all the nodes from the set of reference transcripts that are connected to at least one node from the set of original transcripts and at least one node from the set of compressed transcripts. Let A_i be the original transcript with the highest score compared to the reference node, and let B_i be the compressed transcript with the highest score compared to the reference transcript. The final score is the average of the transcript scores for every pair A_i, B_i.

Tables 10 and 11 show the tripartite scores alongside the percentage of transcripts from the original and compressed set of transcripts that contribute to the score.

Supplementary Table 10. Tripartite score for Cufflinks transcripts.

Dataset	Strict			Loose		
	Score	% True	% Comp	Score	%True	% Comp
Drosophila, Simulated Unpaired						
0.5M	0.999	27.5	27.6	0.981	43.7	43.8
1M	0.999	23.3	23.4	0.986	39.4	39.6
2.5M	0.998	24.9	24.9	0.992	34.7	34.7
5M	0.996	23.9	24.0	0.994	30.6	30.7
10M	0.992	23.1	23.1	0.990	28.0	28.1
20M	0.988	22.3	22.4	0.986	26.9	26.9
Drosophila, Simulated Paired						
0.5M	0.994	45.2	45.2	0.984	59.2	59.2
1M	0.990	35.5	35.5	0.983	47.1	47.1
2.5M	0.979	31.5	31.3	0.977	39.4	39.2
5M	0.979	28.0	27.9	0.976	34.4	34.4
10M	0.969	25.2	25.2	0.963	31.6	31.6
20M	0.965	23.3	23.3	0.959	29.5	29.5
Human, Simulated						
20M	0.976	16.8	16.9	0.972	22.6	22.7
40M	0.976	14.4	14.5	0.970	20.2	20.3

Supplementary Table 11. Tripartite score for Stringtie transcripts.

Dataset	Strict			Loose		
	Score	% True	% Comp	Score	%True	% Comp
Drosophila, Simulated Unpaired						
0.5M	1.000	43.2	43.2	1.000	59.1	59.1
1M	1.000	44.0	4.0	1.000	58.3	58.3
2.5M	0.999	40.9	40.9	0.997	51.1	51.1
5M	0.998	34.3	34.3	0.998	42.3	42.3
10M	0.993	29.4	29.4	0.994	36.4	36.4
20M	0.988	24.5	24.5	0.990	32.0	32.0
Drosophila, Simulated Paired						
0.5M	1.000	44.6	44.7	0.999	58.5	58.6
1M	0.999	41.0	41.0	0.994	51.9	52.0
2.5M	0.996	35.1	35.0	0.996	42.7	42.7
5M	0.995	28.5	28.6	0.994	36.2	36.2
10M	0.989	25.0	25.0	0.988	32.5	32.6
20M	0.986	23.0	23.1	0.986	30.3	30.3
Human, Simulated						
20M	0.985	16.7	16.6	0.985	22.4	22.4
40M	0.983	14.0	14.0	0.981	19.9	19.9

SUPPLEMENTARY NOTE 11

Transcript quantification with Cufflinks and StringTie.

We measured Boiler's effect on quantification accuracy when transcripts are quantified directly from alignments without an initial assembly step. We ran Cufflinks and StringTie in quantification-only mode (`-G` for Cufflinks and `-G -e` for StringTie). As input we use the alignment BAM files output by TopHat 2 in each of the simulation experiments described in the main text. We ran each tool on the BAM file both before and after Boiler compression. For comparison, we also ran each tool on each of the 5 technical replicates described in the "Fidelity" section in the main text. The reference transcriptome provided to each tool was the same one used to generate the original dataset with Flux Simulator. Both tools output a file called "isoforms.fpkm_tracking" with FPKM estimates for all transcripts. Table 12 presents the Root Mean Squared Error (RMSE) between the FPKM vectors before and after Boiler compression (column: "Boiler"). We also obtained 10 pairwise RMSEs by comparing all pairs of technical-replicate FPKM vectors. Minimum and maximum technical-replicate RMSEs are shown in the "Tech Reps" column.

Figure 3 plots Boiler-compressed vs. original FPKM for all transcripts in the StringTie quantitation output for the *D. melanogaster* 20M paired-end dataset (blue points). We also plot the same for a randomly-chosen pair of technical replicates (red points).

While the Boiler RMSE for Cufflinks quantitation is often (though not always) higher than the highest observed for the technical replicates, the Boiler RMSE for StringTie quantitation is always lower than the lowest observed for the technical replicates. This is likely because Boiler removes read names during compression, breaking the relationship between a multi-mapping read and its several alignments. This in turn affects Cufflinks quantification, which depends on read names to group multi-mapping alignments during quantification. A subject for future work is whether and how Boiler can preserve enough information about multi-mapping alignments to control RMSE without substantially decreasing compression ratio.

Note that these technical replicates are simulated, and so exhibit less inter-sample variability than real-world replicates.

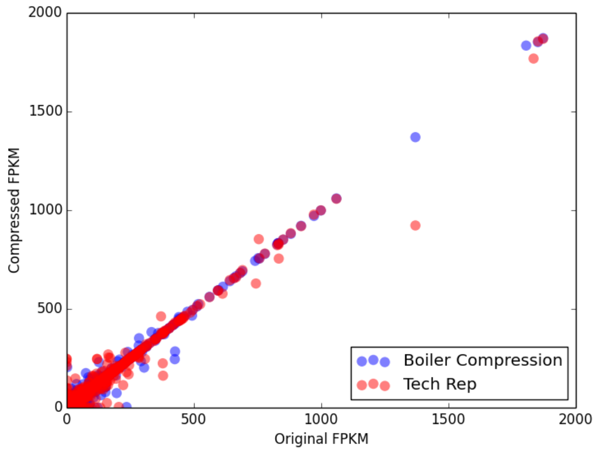

Supplementary Figure 3. Accuracy of quantification results computed with Stringtie.

Supplementary Table 12. RMSE of Quantification Results

Dataset	Cufflinks		StringTie	
	Boiler	Tech Reps	Boiler	Tech Reps
Drosophila, Simulated Unpaired				
0.5M	19.37	16.28–20.39	0.04	15.24–17.27
1M	28.04	11.72–15.10	0.02	12.97–13.78
2.5M	21.12	7.02–9.58	0.06	7.85–9.34
5M	30.36	4.60–5.99	0.85	6.24–11.37
10M	21.87	3.24–4.25	0.13	5.40–7.60
20M	18.07	2.20–2.52	0.19	4.56–8.74
Drosophila, Simulated Paired				
0.5M	8.39	11.96–14.59	0.86	13.25–14.97
1M	4.97	9.14–11.21	0.68	9.28–12.36
2.5M	13.92	5.66–6.88	0.86	7.38–8.96
5M	10.6	3.53–4.22	0.55	5.88–9.27
10M	11.33	2.62–3.37	1.13	5.26–7.89
20M	13.73	1.83–3.02	1.06	5.21–6.76
Human, Simulated				
20M	5.86	0.57–0.81	1.39	3.37–4.16
40M	2.76	0.37–0.47	1.09	4.12 – 5.93

REFERENCES

1. Caprara A, Kellerer H, Pferschy U (2000) A PTAS for the multiple subset sum problem with different knapsack capacities. *Information Processing Letters*, **73(3)**, 111-118.
2. Garey MR, Johnson DS (1978) “Strong” NP-Completeness Results: Motivation, Examples, and Implications. *Journal of the ACM*, **25(3)**, 499–508.
3. Li B, Fillmore N, Bai Y, Collins M, Thomson J, Stewart R, Dewey C (2014) Evaluation of de novo transcriptome assemblies from RNA-Seq data. *Genome Biology*, **15(12)**, 553.
